# The Effectiveness and Relevance of the Canadian Triage System at Times of Overcrowding in the Emergency Department of a Private Tertiary Hospital: A United Arab Emirates (UAE) Study

**DOI:** 10.7759/cureus.52921

**Published:** 2024-01-25

**Authors:** Laila Kalan, Racha A Chahine, Chafika Lasfer

**Affiliations:** 1 Trauma and Orthopaedics, University Hospitals Birmingham National Health Service (NHS) Foundation Trust, Birmingham, GBR; 2 Quality and Risk Management, Fakeeh University Hospital, Dubai, ARE; 3 Emergency Medicine, Fakeeh University Hospital, Dubai, ARE

**Keywords:** uae, five-level triage, ctas, emergency medicine, triage

## Abstract

Objective: A systematic and straightforward triage system is crucial for the proper and timely care of patients within the emergency department (ED). This study unfolds a detailed understanding of the impact of the Canadian Triage and Acuity Scale (CTAS) on patient care and resource allocation in a private tertiary hospital. To the best of our knowledge, this is the only article studying the impact of the CTAS in one of the private hospitals in the United Arab Emirates (UAE) to achieve triage optimisation strategies. There is scope for further research in both public and private hospitals in the UAE. A triage system not only helps healthcare professionals prioritise cases conveniently but also guides patients to the most suitable area for a consultation. As a general rule, EDs follow an algorithm for the purpose of triage, and the aim of our study is to assess one such five-level triage system, CTAS, for its effectiveness and relevance during overcrowding in a UAE ED.

Method: Within a period of approximately three weeks, a total of 351 CTAS-triaged patients were included in a prospective observational study during peak hours (17:00-22:00) of an ED in the UAE. The CTAS app was used as the triage tool to assess relevance, in terms of patient waiting times, resource allocation, and urgency level distribution, to the Canadian scale. All patients presenting to the ED were included with no exclusion criteria. The relationship between urgency level, duration of visit, and resources used was assessed, and the department's triage results were compared with those of the CTAS app.

Results: Our sample showed a female (187; 53.3%) and adult preponderance (215; 61.3%) with most of the adult patients aged between 30 and 40 (96; 44.65%). 41.5% (145) of the triage was mismatched between the department and the CTAS app with 115 (79.3%) cases of under-triaging and 30 (20.7%) cases of over-triaging. There was a statistically significant difference (p=0.004) between average waiting times across triage categories 4 and 5 with the former category patients waiting for a longer period of time. Cohen's kappa showed moderate inter-relatability (k=0.42). The average utilisation costs per triage category showed a positive correlation with the urgency level for CTAS (Pearson's r=0.59); however, the costs declined as the urgency level rose for the department.

Conclusions: The high compliance rate demonstrates that the CTAS can be applicable to institutions outside of Canada. The categorisation of patients by the CTAS and their resource allocation were more accurate than the standard triage proving its effectiveness as a triage tool. Lack of synchronisation among the triage nurses and inadequate triage training are the most plausible reasons for this comparison. The recommended "time to be seen by a physician" was achievable in our ED, and that, along with the expected relationship between CTAS and resource utilisation, can be seen as valid indicators for a quality triage system for use in the UAE.

## Introduction

In the ever-evolving landscape of emergency healthcare, an efficient triage system stands as a linchpin for providing timely and prioritised care. The Canadian Triage and Acuity Scale (CTAS), a widely acknowledged five-level triage system, has demonstrated efficacy in optimising patient flow and resource utilisation in various settings. It has gained recognition for its effectiveness in various healthcare settings, including Canada, Australia, and the United Kingdom [1-3]. Its ability to accurately categorise patients and guide resource allocation has made it a valuable tool in emergency departments (EDs). However, the application of such a system in diverse international contexts, particularly within the dynamic healthcare environment of the United Arab Emirates (UAE), necessitates careful scrutiny.

A comprehensive review of the literature reveals that CTAS implementation has been associated with a range of positive outcomes, including reduced waiting times, improved patient satisfaction, and decreased length of stay in EDs [2,4]. Moreover, CTAS has demonstrated effectiveness in optimising resource utilisation, ensuring that patients receive care promptly and according to their needs [4,5].

The applicability of CTAS in non-Canadian settings has also been extensively evaluated. Studies conducted in Australia and the United Kingdom have shown that CTAS can be effectively integrated into existing triage systems, leading to improvements in patient flow, resource management, and overall quality of care [6,7].

The latest evidence further supports the effectiveness of CTAS in enhancing patient outcomes and resource utilisation in EDs worldwide. A recent study by Al-Qahtani et al. this year [8] found that CTAS implementation in a Saudi Arabian ED resulted in a significant decrease in waiting times for private patients with urgent and non-urgent conditions. Similarly, a study by Al Yasin et al. [9] evaluated the feasibility and validity of the CTAS in EDs by examining specific quality indicators, such as waiting times and length of stay. Findings underscore the impact of a fast-track system in improving patient flow for non-urgent cases. Our study investigates not only patient waiting times but also resource allocation for each triage category assigned by CTAS. These findings underscore the versatility and effectiveness of CTAS as a triage tool, making it a valuable asset for improving emergency care delivery across diverse healthcare settings.

This study delves into the contextual relevance and effectiveness of the CTAS in an ED setting in the UAE, especially during periods of overcrowding. As EDs grapple with escalating patient volumes, the need for a nuanced understanding of triage dynamics becomes increasingly critical. By assessing the performance of the CTAS, we aim to discern its alignment with departmental triage outcomes, its impact on patient waiting times, and its implications for resource utilisation.

Through a prospective observational study involving 351 CTAS-triaged patients, we navigate the intricacies of private emergency care in the UAE. The study not only scrutinises the triage outcomes but also explores the relationship between urgency levels, visit durations, and associated resource use. The findings promise to contribute valuable insights into enhancing emergency care practices in the UAE, shedding light on the adaptability and effectiveness of the CTAS beyond its Canadian origins.

## Materials and methods

We conducted a prospective observational study, gathering data from all individuals presenting to the ED of Fakeeh University Hospital in Dubai, UAE, during peak hours, specifically between 17:00 and 22:00. Observation before the study showed a busier ED with higher patient turnover during these five hours. Over a three-week period in December 2022, we collected 351 data entries. The CTAS app was employed to categorise patients based on their urgency levels.

**Table 1:** The five-level Canadian Triage and Acuity Scale

Canadian Triage and Acuity Scale		
Level 1	Resuscitation	Threat to life or limbs
Level 2	Emergent	Potential threat
Level 3	Urgent	Potential progression of state
Level 4	Less urgent	Potential for deterioration
Level 5	Non-urgent	Intervention can be delayed

To assess the effectiveness of the triage system, the time interval between registration, triage, and being seen by a physician was noted. Patient factors that could possibly affect the duration of the visit were added to the data set, such as age, gender, nationality, presenting symptoms, etc. A total of three nurses were responsible for triage. The characteristics of the study are presented as either a percentage, N, or the mean±median.

A comparison was made between the categories and their implications assigned by the hospital's triage system and those of the CTAS to determine whether or not the Canadian triage guidelines were met. We used a single-factor analysis of variance (ANOVA) test to compare the "time to be seen by a physician" between different urgency levels within the hospital. A p-value of <0.05 was considered to be statistically significant. Cohen's kappa was used to check the level of agreement between both triage methods.

The Institutional Review Board (IRB) Committee of Fakeeh University Hospital chaired by Dr. Abdelrahman King granted the approval (approval number: FUH-RES-016-023) and IRB exemption validating the adherence of our study to ethical standards. 

## Results

In December 2021, 351 patients were triaged during peak hours in the ED. Data from weekends was omitted due to a lack of data collection on these days. 

**Figure 1:**
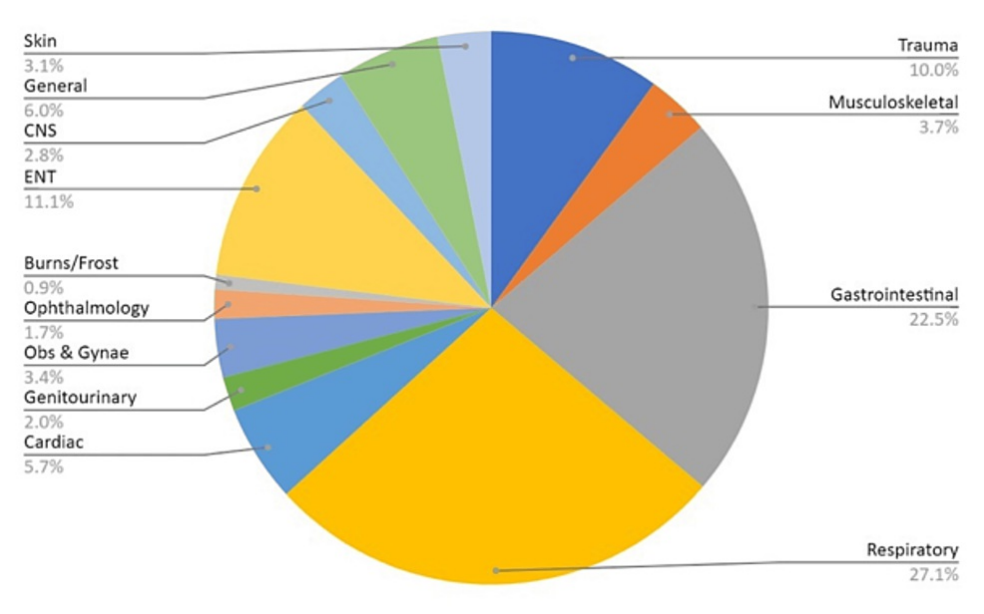
Symptom distribution This pie chart shows different clinical presentations, as a percentage, within the study population.

There was a female preponderance (53.3%, n=187), with the majority of the patients being adults (61.3%, n=215) within the 30-40-year-old age group (44.65%, n=96). Most of the patients were of South Asian origin (33.6%, n=118), and the most common presentations were respiratory (27.1%, n=95) followed by gastrointestinal symptoms (22.5%, n=79) (Figure 1). It is worth mentioning that 26.5% (n=93) of those who presented to the ED complained of flu-like symptoms and fever. Wednesdays and Thursdays saw the greatest number of patients at 27 and 25, respectively. The patient demographics are presented in Table 2. No statistically significant difference was found in the demographics. 

**Table 2:** Patient demographics The data has been represented as N (%).

Demographics		
Gender/age (years) (n=351)	<18 (n=136) (%)	>18 (n=215) (%)
Male (n=164)	71 (52%)	93 (43%)
Female (n=187)	65 (48%)	122 (57%)

**Figure 2:**
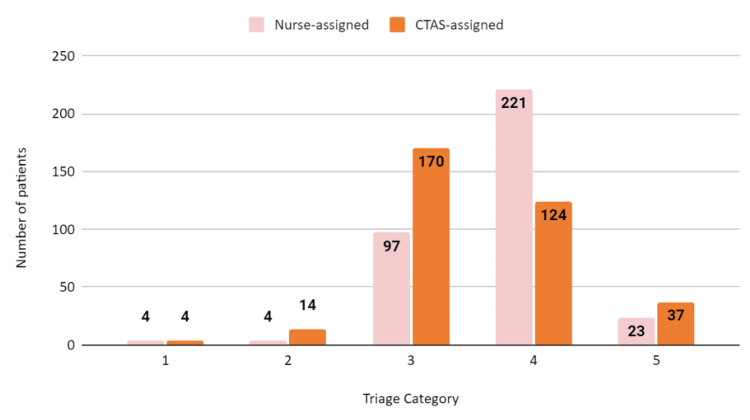
Assignment of triage categories by nurses and the CTAS app The data has been represented as N. CTAS: Canadian Triage and Acuity Scale

The assignment of triage categories by the nurses and the app were compared. Two patients were excluded from the triage comparison analysis as they bypassed the triage process by directly approaching physicians. The following bar graph represents the comparison of urgency levels assigned by both methods (Figure 2).

According to the tertiary hospital's protocol, more than half of the patients (63%, n=221) were categorised as level 4, whilst the CTAS app categorised 48.5% (n=170) patients as category 3 and only 124 patients (35.4%) as level 4. Twenty-seven percent more patients were assigned level 4 by the nurses. The results were the same for level 1, and slight discrepancies were noted in the other categories.

The triage results for the subjects showed a 41.5% (n=145) mismatch, out of which 115 (79%) patients were under-triaged and 30 (21%) cases were over-triaged. Cohen's kappa for the level of agreement for triage categories 3 and 4 by both methods was moderate at k=0.42.

The compliance rate of nursing triage to the CTAS triage was 45% and 53% for the respiratory and cardiac symptoms, respectively. The ratio of the cardiac patients assigned by the nurses to the app for category 2 was 1:6.

**Figure 3:**
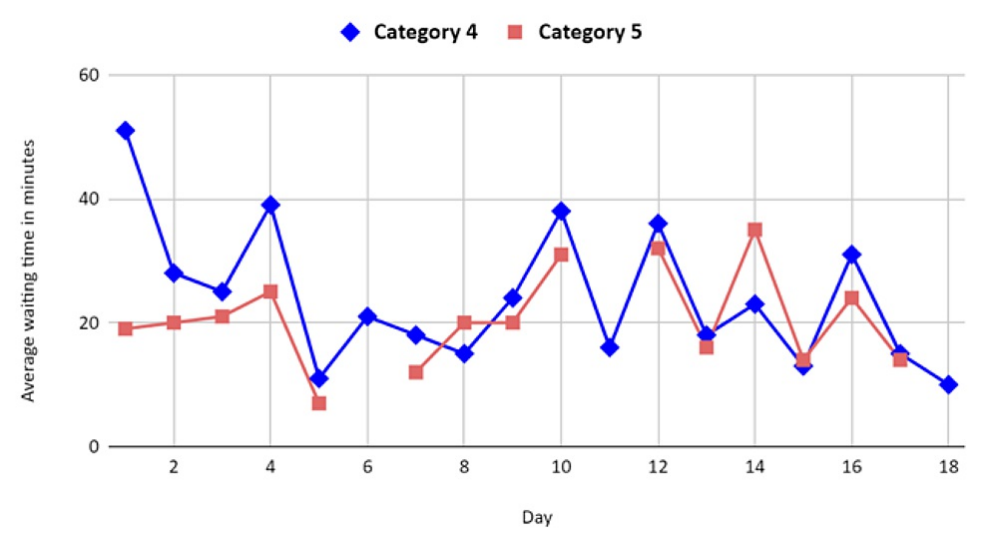
Average waiting time across triage categories 4 and 5 per day The data has been represented as mean. A p-value of <0.05 was considered significant.

The average waiting time over the study duration was 22 minutes (Figure 3). The mean waiting time, in minutes, for the less urgent triage categories were 20.5 (category 3, median=21.5), 24 (category 4, median=22), and 20.6 (category 5, median=20). A single-factor ANOVA test carried out to compare the average waiting times for categories 4 and 5 showed a statistically significant difference with a p-value of 0.004. Overall, category 4 patients waited longer than category 5 patients for 80% of the study duration. No statistically significant difference was noted in the other categories. 

Thirteen percent (n=45) of the cases exceeded the "time to be seen by a physician" recommendation with no differences noted between the adult and paediatric populations. However, slightly more females made up the 13%, and the majority of the symptoms were respiratory.

**Figure 4:**
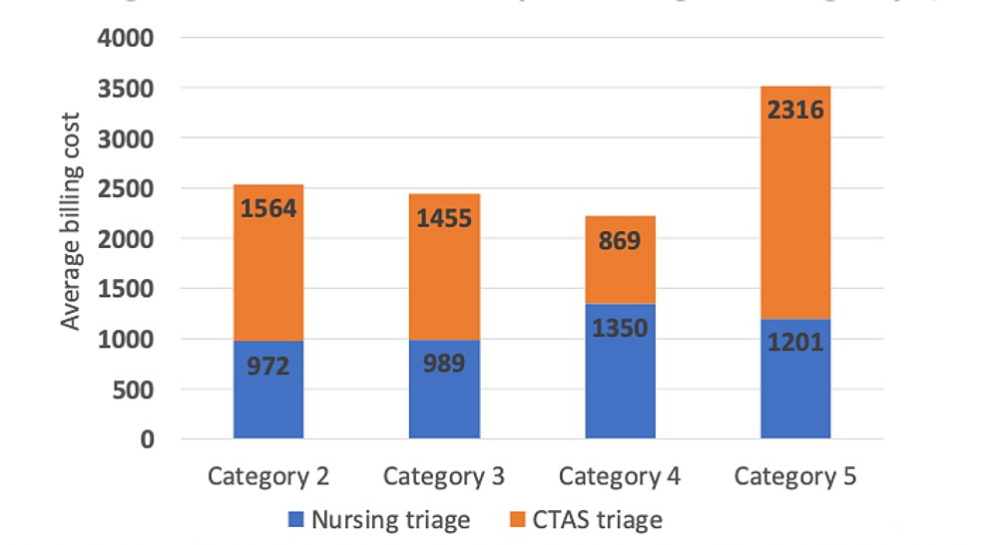
Average utilisation costs per triage category for both triage methods The data has been represented as mean.

A comparison of the average utilisation costs per triage category showed a moderate positive correlation (Pearson's r=0.59) with increasing urgency for the CTAS method and a negative correlation with the nursing method, excluding the least urgent category 5 (Figure 4).

## Discussion

In substantiating the efficacy of the CTAS within the ED context, this study unfolds a nuanced understanding of its impact on patient care and resource allocation. Overcrowding can result in difficult decision-making and negatively impact quality of care [10-12]. Therefore, a systematic triage system is vital in dealing with high patient numbers. The impressive 93% compliance rate observed in the "time to be seen by a physician" metric attests to the system's efficacy in expediting patient assessment and management, aligning with the fundamental goals of efficient emergency care. This allows for timely patient management, including earlier recognition of red flags and, as a result, provision of earlier specific treatment. As for the ED, the high compliance rate means a high patient turnover and flow within the department which is advantageous for staff and patients alike.

A noteworthy aspect of the study is the moderate level of agreement discerned between nursing triage and the CTAS system. This finding not only underscores the reliability of CTAS across distinct stages of patient evaluation but also emphasises its validity in effectively triaging patients with diverse acuity levels. This level of consensus could be due to factors such as the presence and regularity of triage training and the number of triage nurses and their understanding of the hospital's digital triage platform. Depending on the outcome of these factors, the reliability of the triage system may be positively or negatively affected. This agreement, though moderate, sheds light on the consistent application of the CTAS framework, offering reassurance in its applicability across varied patient scenarios.

Delving deeper into the temporal analysis of triage categories, the study reveals an intriguing pattern of prolonged wait times for category 4 patients compared to their category 5 counterparts. This temporal nuance, witnessed during 80% of the study duration, highlights a specific area for potential enhancement. Recognising and understanding such temporal variations are pivotal for refining processes and introducing targeted interventions to streamline the treatment of semi-urgent cases.

The demographic insights presented through Figures 1-3 add a layer of complexity and strategic insight to the study. These figures not only elucidate the distribution of urgency levels across different age groups but also underscore the prevalence of CTAS 3 and CTAS 4 classifications, particularly in the 0-4 age group. Moreover, the gender-based distribution reaffirms the predominance of CTAS 3 and CTAS 4 across both male and female patient groups, signalling the need for a nuanced exploration of potential gender-specific patterns influencing triage outcomes.

The correlation between CTAS category and waiting time, illustrated in Figure 4, underscores a critical aspect of the triage system: its role in determining resource allocation and patient flow. Notably, the prolonged wait times for category 4 patients emerge as a focal point, urging the consideration of targeted interventions to optimise the management of semi-urgent cases and, in turn, enhance overall ED efficiency.

In light of these findings, it is crucial to acknowledge the economic implications of triage categories, an aspect not explicitly addressed in the study. As inferred, higher average costs are associated with CTAS 2 patients, a fact that adds a layer of complexity to the resource allocation and budgeting considerations within the ED. This economic perspective, though a tangent to the primary focus, remains crucial for informed decision-making and aligning financial considerations with the acuity levels and resource requirements of diverse patient categories.

Triage nurses need to be thoroughly aware of patient conditions and their acuity in order to prioritise them accordingly. Urgent and accurate categorisation of patients is pivotal in quality patient care. A study conducted by Bahlibi et al. in 2022 [13] demonstrated only 3% have adequate knowledge about triaging with a mean knowledge score of 6.23. Mean knowledge scores after training and after a three-month follow-up were 10.55 and 9.39, respectively. This stresses the importance of staff education for a successful triage system. Knowledge degradation over time also emphasises the incorporation of regular triage training and supervision programs [13,14]. Factual knowledge is crucial in making triage decisions and, as a result, improving patient outcomes [15-18]. Overall, this approach not only introduces objectivity but also provides a consistent set of data.

Further research is warranted to explore the long-term impact of CTAS implementation on patient outcomes and resource utilisation, particularly in specific patient populations such as children and older adults. Additionally, investigations into factors that influence the accuracy and reliability of CTAS implementation can provide valuable insights for optimising its effectiveness in various settings. Moreover, there is scope for further studies on discharge and admission waiting times to assess patient flow between various departments. The reasons behind delayed transfers, such as exit blocks and boarding [19,20], can be sought to further aid in the formulation of optimisation strategies. Furthermore, research can be extended into both public and private hospitals to compare the effectiveness of the CTAS in both environments. 

In terms of limitations, drawing conclusive findings from a brief three-week study period may not be optimal for establishing robust generalisations. A more extended study duration would not only enhance the study's validity but also allow for a more substantial sample size, thereby bolstering overall reliability. The exclusion of weekends introduces non-continuous data, further limiting the study's capacity for broader generalisation.

Additionally, the study's reliance on a restricted number of triage nurses raises concerns about potential subjectivity in the results. The limited diversity among triage practitioners could influence compliance rates, thereby introducing a variable that may impact the overall study outcomes. Recognising these limitations is crucial for a nuanced interpretation of the findings. Future research endeavours should consider extended study durations and a more varied pool of triage personnel to address these constraints, fortifying the study's methodological robustness.

## Conclusions

The study robustly validates the efficacy and adaptability of the CTAS within the challenging environment of the ED in the UAE. The notable 93% compliance rate in the "time to be seen by a physician" metric underscores the system's replicability and scalability, positioning it as a feasible model for adoption in analogous settings. Despite a 53% compliance rate to CTAS categories, the inherent relativity of this metric necessitates nuanced interpretation, emphasising the need for a comprehensive understanding of triage dynamics.

The study's identification of areas for enhancement, particularly the addressed delays in category 4 patients, serves as a catalyst for targeted interventions aimed at refining patient care and optimising resource utilisation. The multifaceted approach outlined encourages other healthcare facilities to reassess their processes, leveraging the demonstrated success of CTAS to minimise delays in semi-urgent cases. Moreover, the demographic insights offer a strategic template for tailoring interventions and allocating resources based on age and gender-specific patient needs. The study's pivotal role in establishing achievable standards for triage systems transcends institutional boundaries, guiding the formulation of best practices applicable across diverse healthcare settings. As we contemplate the broader implications, the comparative effectiveness of CTAS against alternative triage systems emerges as a key consideration for advancing ED operations, beckoning further exploration into comparative analyses and collaborative efforts to refine and implement the CTAS model across diverse healthcare landscapes.
